# Mechanical ventilation modes for respiratory distress syndrome in infants: a systematic review and network meta-analysis

**DOI:** 10.1186/s13054-015-0843-7

**Published:** 2015-03-20

**Authors:** Changsong Wang, Libo Guo, Chunjie Chi, Xiaoyang Wang, Lei Guo, Weiwei Wang, Nana Zhao, Yibo Wang, Zhaodi Zhang, Enyou Li

**Affiliations:** Department of Anesthesiology, First Affiliated Hospital of Harbin Medical University, No 23 Youzheng Str, Nangang District, Harbin, Heilongjiang 150001 China; Department of Implantology, Hospital of Stomatology, Harbin Medical University, No 23 Youzheng Str, Nangang District, Harbin, Heilongjiang 150001 China; Department of Anesthesiology, The Third Affiliated Hospital of Harbin Medical University, No 150 Haping Str, Nangang District, Harbin, Heilongjiang 150001 China

## Abstract

**Introduction:**

The effects of different mechanical ventilation (MV) modes on mortality outcome in infants with respiratory distress syndrome (RDS) are not well known.

**Methods:**

We searched the Cochrane Central Register of Controlled Trials (CENTRAL) in the Cochrane Library, EMBASE, MEDLINE, CINAHL, and Web of Science for studies published through April 2014 that assessed mortality in infants with RDS given different MV modes. We assessed studies for eligibility, extracted data, and subsequently pooled the data. A Bayesian fixed-effects model was used to combine direct comparisons with indirect evidence. We also performed sensitivity analyses and rankings of the competing treatment modes.

**Results:**

In total, 20 randomized controlled trials were included for the network meta-analysis, which consisted of 2,832 patients who received one of 16 ventilation modes. Compared with synchronized intermittent mandatory ventilation (SIMV) + pressure support ventilation (PSV), time-cycled pressure-limited ventilation (TCPL) (hazard ratio (HR) 0.290; 95% confidence interval (CI) 0.071 to 0.972), high-frequency oscillatory ventilation (HFOV) (HR 0.294; 95% CI 0.080 to 0.852), SIMV + volume-guarantee (VG) (HR 0.122; 95% CI 0.014 to 0.858), and volume-controlled (V-C) (HR 0.139; 95% CI 0.024 to 0.677) ventilation modes are associated with lower mortality. The combined results of available ventilation modes were not significantly different in regard to the incidences of patent ductus arteriosus and intraventricular hemorrhage.

**Conclusion:**

Compared with the SIMV + PSV ventilation mode, the TCPL, HFOV, SIMV + VG, and V-C ventilation modes are associated with lower mortality.

**Electronic supplementary material:**

The online version of this article (doi:10.1186/s13054-015-0843-7) contains supplementary material, which is available to authorized users.

## Introduction

Respiratory distress syndrome (RDS) is a common clinical disease that results from the deficiency of alveolar surfactant along with the structural immaturity of the lungs in preterm infants [1]. EuroNeoNet figures in 2010 indicated RDS rates of 92% at 24 to 25 weeks, 88% at 26 to 27 weeks, 76% at 28 to 29 weeks, and 57% at 30 to 31 weeks of gestation [2]. RDS remains the primary cause of infant mortality [3].

The European Consensus Guidelines [1] for 2013 recommend that non-invasive respiratory support be used at birth for all infants at risk for RDS, thereby avoiding a greater chance of mechanical ventilation (MV) [4]. However, non-invasive ventilation cannot always provide effective oxygenation and stable lung mechanics [5]. Therefore, MV remains an essential and life-saving technique to care for preterm infants with RDS for whom non-invasive ventilation fails [1].

Research has sought to develop ventilation that avoids the development of lung injury and consequent bronchopulmonary dysplasia (BPD) as well as decreases the mortality of preterm infants. However, the conclusions associated with the efficacy and safety of these ventilation techniques remain controversial [6,7], highlighting the potential significance of the optimal ventilation mode for preterm infants with RDS. Therefore, researchers have attempted to identify the optimal ventilation mode for infants with RDS via meta-analyses [8]. However, traditional meta-analyses can compare only two treatments (or classes) that have been compared in head-to-head trials [9]. The MV of preterm infants with RDS contains many modes, including assist/control (A/C) ventilation [10], high-frequency oscillatory ventilation (HFOV) [7], volume controlled (V-C) ventilation [11], and volume-guaranteed (VG) ventilation [12]. Therefore, the ability to draw definitive conclusions from the results of traditional meta-analyses is limited. A network meta-analysis enables the evaluation of the comparative effectiveness of multiple interventions even though certain pairs might not be directly compared. The idea that underlies network meta-analysis methodology for a given comparison between two treatments A and B is that direct evidence (which originates from studies that compare A with B) and indirect evidence (which originates from the combination of studies through an intermediate comparator, for example, A versus C and B versus C studies) can be synthesized into a single effect size. Furthermore, this analysis has the potential to reduce the uncertainty in treatment effect estimates [13]. However, certain methodological aspects are poorly understood, and there are challenges in the application and interpretation of data synthesis. This method is not perfect and poses various challenges; for example, both conceptual and statistical heterogeneity and incoherence between included studies should be carefully assessed. Furthermore, estimates of treatment effects should be interpreted with attention to their uncertainty; though appealing, plain treatment rankings or probabilities derived from network meta-analyses can be misleading [14]. In this study, we attempted to provide suggestions for the treatment of infants with RDS by taking advantage of a network meta-analysis.

## Methods

We conducted our systematic review in accordance with the methods recommended by the preferred reporting items for systematic reviews and meta-analyses (PRISMA) guidelines [15].

### Literature search

The trials were identified via electronic and manual searches. We searched the Cochrane Central Register of Controlled Trials (CENTRAL) in the Cochrane Library, EMBASE, MEDLINE, CINAHL, and Web of Science by using a combination of Medical Subject Headings (MeSH) and text words (Additional file 1). We did not restrict our search by language or year of publication. The last search update was performed in April 2014. We reviewed the reference lists of the published meta-analyses. In addition, we manually searched the Index Medicus for randomized controlled trials (RCTs), meta-analyses, and systematic reviews for studies that the initial electronic search missed.

### Literature inclusion and exclusion

Two groups independently assessed whether the literature reports should be included in the study analyses. To resolve discrepancies between the decisions of these two groups, the groups met, discussed, and jointly decided whether a disputed report should be included. We first used EndNote X6 to identify duplicate publications. We subsequently removed reviews, retrospective studies, observational studies, case reports, experimental studies of adults, animal studies, research that addressed physiological mechanisms only, irrelevant studies (such as studies of MV in patients who did not suffer from neonatal RDS, studies of pulmonary surfactant treatments, and therapeutic approaches other than MV for infants with RDS), duplicate reports, duplicate experiments (such as assessments of other studies and reports that described secondary or *post hoc* analyses of previously published experimental data), and research that addressed non-invasive MV. After the full texts of the remaining reports were obtained and carefully read, non-randomized trials and experiments that used a crossover design were excluded. Finally, RCTs of MV for infants with RDS were included in the current analyses. All studies included were of high quality and had a low risk of bias; therefore, no publications were excluded on the basis of research quality assessments.

### Outcome measures and data extraction

The information extracted from the analyzed literature included study-specific data (such as experimental design, inclusion criteria, and the time and location of the experiments), information in regard to the selected infants (such as gestational age and birth weight), the specific processes used to conduct the experimental research, the MV modes, and the clinical and safety outcomes in regard to the infants with RDS. The primary outcome of the current study was the mortality of the infants with RDS; if an included investigation provided multiple mortality rates, then the mortality rate associated with the longest follow-up period was analyzed. The secondary outcomes of the current study included pneumothorax, BPD, intraventricular hemorrhage (IVH) (grade of at least III), patent ductus arteriosus (PDA), length of intensive care unit (ICU) stay, and length of hospital stay. IVH grading was performed by using the scale of Papile *et al*. [16]. Two groups extracted data from the included studies; these data sets were subsequently compared and verified. When necessary, data extractions were sent to the original authors or reporters for data supplementation and correction. We also contacted the authors of certain publications for their assistance with missing or questionable data.

### Statistical analyses

Direct and indirect evidence from all relevant studies was integrated by using a network meta-analysis, and estimates with maximum power were provided [17]. A network meta-analysis was performed by using the GeMTC package in R (i386 3.0.2) [18]. Different timespans were used to calculate mortality across the included studies; therefore, to achieve the maximum accuracy and effectiveness [19], the current investigation used hazard ratio (HR) values and 95% confidence intervals (CIs) as approximations to measure mortality in infants with RDS who received MV [20]. The statistical analysis is based on Poisson likelihoods with a log link function. Odds ratios (ORs) and 95% CIs were used to measure the incidences of pneumothorax, IVH (grade of at least III), and BPD in infants with RDS. The statistical analysis was based on binomial likelihoods with a logit link function. Mean differences (MDs) and CIs were used to analyze the continuous variables of MV duration and length of ICU stay. CIs were calculated by using a Bayesian fixed-effects model that simulated the methods of traditional probability theory. Cases in which the CIs did not include 1.0 were considered significant. We performed Bayesian analyses by using a Markov chain Monte Carlo simulation to calculate the HRs, ORs, MDs, and CIs [17].

Model selection was based on the guidelines of Dias *et al*. [21] for the evaluation of linear models. Dbar indicates the posterior mean of the residual deviance. pD indicates the effective number of parameters (leverage). DIC indicates the ‘deviance information criterion’. A smaller Dbar value indicates a better model fit. However, the model with the lowest DIC is generally chosen to aid interpretation because it accounts for model complexity. A lower DIC value indicates a better model fit. Differences of less than 3 to 5 between the models were not considered significant [22]. A fixed-effect model (Additional file 2) with HRs was chosen for the combined effect size for mortality. A fixed-effect model (Additional files 3 and 4) with ORs was chosen as the combined effect sizes for the incidences of PDA and IVH (grade of at least III). The simulation generated 150,000 iterations, and convergence was assessed by using the Brooks-Gelman-Rubin diagnostic [23]. We used the ‘back-calculation’ [24] technique to evaluate the findings of the network meta-analysis that originated from the direct versus indirect evidence for consistency. Within this analysis, three types of models were estimated: unrelated study effects, unrelated mean effects, and consistency.

The output of the summary function was plotted for visual inspection. Forest plots and the I^2^ statistic were used to investigate the possibilities of statistical heterogeneity and inconsistency between the direct and indirect effect estimates by using the Higgins-Thompson method (low heterogeneity = 25%, moderate heterogeneity = 50%, and high heterogeneity = 75%) [25]. We also ranked the different interventions in terms of their likelihood to obtain the best result for each outcome [26]. Each ventilation mode in the Markov chain Monte Carlo cycle was classified on the basis of an estimated effect size. These probabilities summed to 1 for each treatment and each rank. X% denotes that the mode achieved x% effectiveness; thus, larger percentages denote more effective interventions. However, these percentages indicate possibilities only and are not deterministic [26].

### Sensitivity analyses

For the sensitivity analyses, we eliminated studies by Salvo *et al*. [27] and Castoldi *et al*. [28] from consideration because these studies included participants with gestational ages of 25 to 26 weeks; therefore, the examined populations were significantly different from the participants of the other studies.

## Results

**Figure 1:**
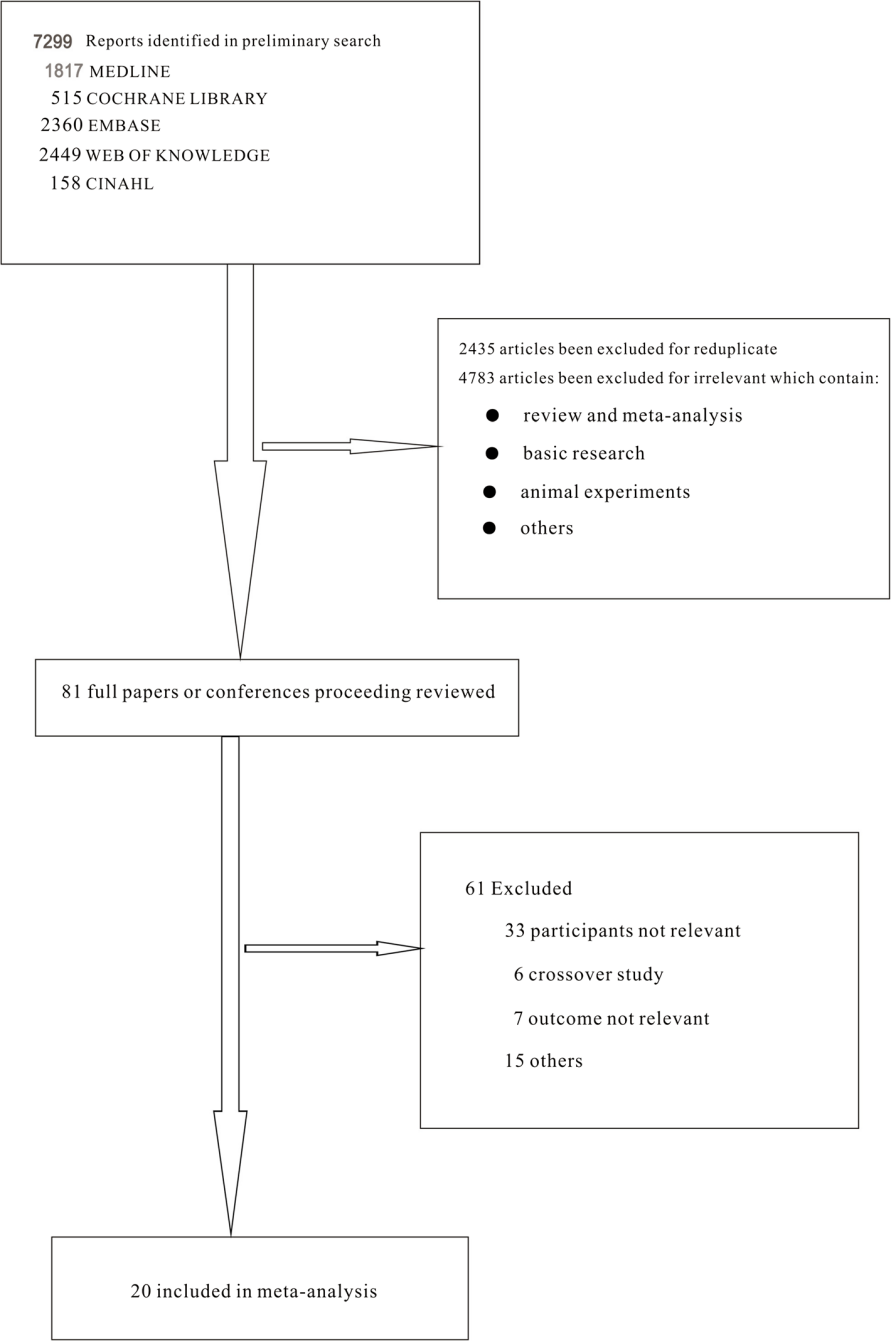
**Flow diagram of the literature search.**

**Figure 2:**
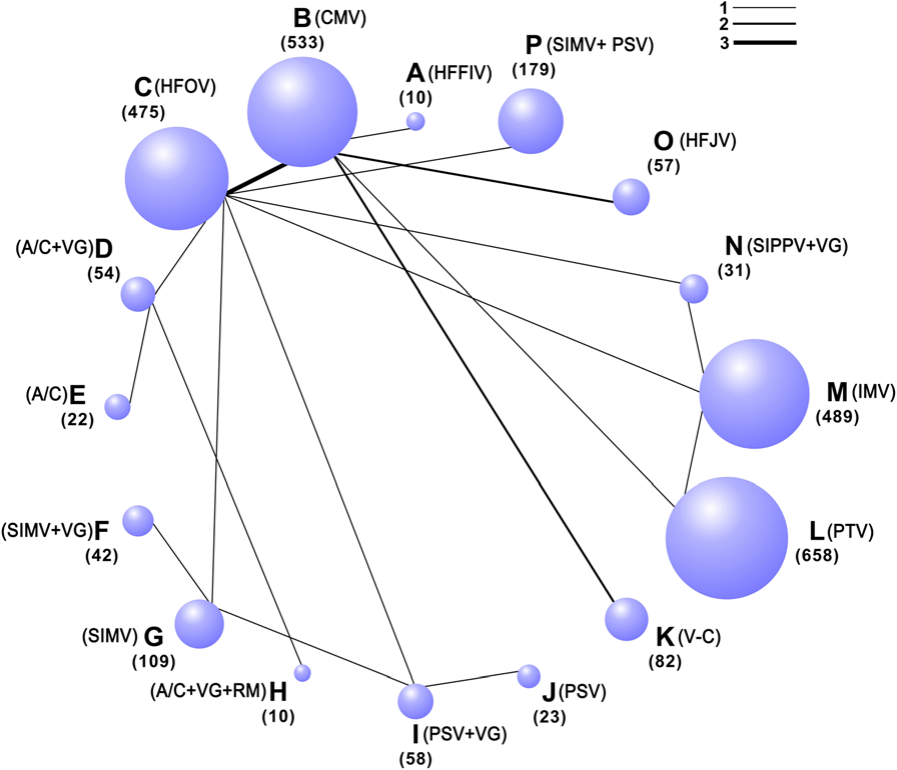
**Network of the comparisons for the Bayesian network meta-analysis.** The size of the nodes is proportional to the number of patients (in parentheses) randomly assigned to receive the treatment. The width of the lines is proportional to the number of trials (next to the line) that compare the connected treatments. A/C, assist-control ventilation; A/C + VG, assist-control plus volume-guarantee ventilation; A/C + VG + RM, assist-control plus volume-guarantee ventilation with recruitment maneuver; CMV, continuous mandatory ventilation; HFFIV, high-frequency flow interrupted ventilation; HFJV, high-frequency jet ventilation; HFOV, high-frequency oscillatory ventilation; IMV, intermittent mandatory ventilation; PSV, pressure support ventilation; PSV + VG, pressure support ventilation with volume-guarantee ventilation; PTV, patient-triggered ventilation; SIMV, synchronized intermittent mechanical ventilation; SIMV + PSV, synchronized intermittent mechanical ventilation with pressure support ventilation; SIMV + VG, synchronized intermittent mechanical ventilation with volume-guarantee ventilation; SIPPV + VG, synchronized intermittent positive pressure ventilation plus volume-guarantee ventilation; V-C, volume-controlled (through adjusting the delivered tidal volume).

We identified 7,299 studies for review on the basis of their titles and abstracts (Figure 1). After an initial screening, we retrieved the full text of 81 potentially eligible articles for a detailed assessment. Finally, we excluded 61 irrelevant articles (Additional file 5), and 20 RCTs were included for network meta-analysis (Table 1), which included 2,832 patients who received one of 16 ventilation modes (Figure 2 and Table 2). All studies were RCTs [6,7,10-12,27-41].

**Table 1:** **Characteristics of the randomized controlled trials of 16 ventilation modes for infants with respiratory distress syndrome**

**Reference**	**Ventilation modes**	**Number of patients**	**Birth weight, grams**	**Gestational age, weeks**	**Quality assessment, Jadad scale**	**Diagnosis**	**Result**						
							**Mortality**	**ICU length of stay, days**	**Pneumothorax, number**	**Hospital length of stay, days**	**BPD, number**	**IVH of at least grade III, number**	**PDA, number**
Singh *et al*. [6]	HFOV vs. SIMV	110	1,398 ± 321/1,393 ± 320	32.0 ± 2.4/31.9 ± 2.5	7	RDS	Yes	No	No	10.3 ± 7.1/10.3 ± 7.1	No	No	No
Lista *et al*. [7]	HFOV vs. A/C + VG	40	1,015 ± 200/1,006 ± 185	27.3 ± 2/27.4 ± 2	6	RDS	Yes	No	No	No	No	1/1	No
Duman *et al*. [10]	A/C + VG vs. A/C	45	1,055.8 ± 236.3/975.5 ± 294.3	27.8 ± 1.7/27.6 ± 2.1	5	RDS	Yes	No	2/2	47.5 ± 22.9/54.2 ± 22.5	3/7	3/7	14/14
Sinha *et al*. [11]	V-C vs. TCPL	50	1,793 ± 513/1,762 ± 503	31.2 ± 2.1/31.2 ± 2.5	5	RDS	Yes	No	0/3	No	1/6	No	4/6
Guven *et al*. [12]	SIMV + VG vs. SIMV	72	1,352.57 ± 373.83/1,275.00 ± 311.63	29.40 ± 2.12/29.17 ± 1.84	7	RDS	Yes	No	No	45.50 ± 31.43/40.00 ± 31.24	2/9	No	No
Salvo *et al*. [27]	HFOV vs. TCPL	88	869 ± 266/913 ± 224	26.4 ± 2.2/26.5 ± 3.2	7	RDS	Yes	21 ± 12/36 ± 23	No	53 ± 21/77 ± 33	No	3/6	No
Castoldi *et al*. [28]	A/C + VG + RM vs A/C + VG	20	747 ± 233/737 ± 219	25 ± 2/25 ± 2	6	RDS	Yes	No	No	No	0/2	No	6/4
Carlo *et al*. [29]	TCPL vs. HFJV	40	1,470 ± 350/1408 ± 240	30 ± 2/30 ± 2	5	RDS	Yes	No	6/3	No	4/3	No	No
HiFO Study Group [30]	HFOV vs. TCPL	176	1,732 ± 979/1,744 ± 853	31 ± 4/31 ± 4	7	RDS	Yes	No	No	No	No	No	24/28
Pardou *et al*. [31]	HFFIV vs. TCPL	22	1,454 ± 197/1,194 ± 23	29.8 ± 1.5/29.2 ± 1.96	5	RDS	Yes	No	5/3	No	No	1/2	No
Wiswell *et al*. [32]	TCPL vs. HFJV	73	930 ± 220/961 ± 314	26.6 ± 2.1/26.9 ± 2.9	7	RDS	Yes	No	7/3	75.9 ± 18.7/66.1 ± 33.8	No	No	No
Gerstmann *et al*. [33]	HFOV vs. TCPL	125	1,560 ± 460/1,460 ± 470	30.8 ± 2.2/30.1 ± 2.7	6	RDS	Yes	No	No	44.0 (30.6,52.6)/46.9 (39.2,56.0)^a^	No	2/6	20/19
Beresford *et al*. [34]	PTV vs. TCPL	386	1,336 (1,000-1,997)/1,320 (1,006-1,996)^c^	29 (25-36)/29 (25-34)^c^	7	RDS	Yes	No	20/21	No	No	11/13	No
Baumer [35]	PTV vs. IMV	924	1,097 ± 327/1,123 ± 345	27.8 ± 2.0/27.8 ± 2.1	6	RDS	Yes	No	62/47	No	No	No	No
Lista *et al*. [36]	PSV + VG vs. PSV	53	1,125 ± 370/1,197 ± 333	28.5 ± 2/29.4 ± 1.6	5	RDS	Yes	No	0/3	No	3/4	No	No
Nafday *et al*. [37]	SIMV vs. PSV + VG	34	1,055 ± 77/1,198 ± 108	27.4 ± 0.5/27.9 ± 0.6	6	RDS	Yes	No	No	No	No	6/3	5/4
Dani *et al*. [38]	HFOV vs. PSV + VG	25	1,126 ± 170/1,075 ± 313	28.3 ± 1.5/28.0 ± 1.3	6	RDS	Yes	66.2 ± 19.9/62.8 ± 24.2	No	66.2 ± 19.9/62.8 ± 24.2	4/3	No	11/9
Singh *et al*. [39]	V-C vs. TCPL	109	985/976^b^	27.1/27.2^b^	7	RDS	Yes	No	2/4	No	No	5/5	17/15
Liu *et al*. [40]	SIPPV + VG vs. HFOV vs. IMV	84	1,702 ± 701/1,813 ± 732/1,902 ± 603	31.5 ± 3.6/30.2 ± 4.8/32.3 ± 3.4	5	RDS	Yes	No	No	No	No	No	No
Sun *et al*. [41]	HFOV vs. SIMV + PSV	356	1,129 ± 199/1,117 ± 241	29.3 ± 2.5/29.5 ± 2.3	7	RDS	Yes	No	10/21	27.0 ± 20.2/31.6 ± 21.7	13/28	No	No

‘Yes’ represents the existence of a result, and ‘No’ refers to no result. ^a^Median (95% confidence limits), ^b^mean, ^c^median (interquartile range). A/C, assist-control ventilation; A/C + VG, assist-control plus volume-guarantee ventilation; A/C + VG + RM, assist-control plus volume-guarantee ventilation with recruitment maneuver; BDP, bronchopulmonary dysplasia; HFFIV, high-frequency flow interrupted ventilation; HFJV, high-frequency jet ventilation; HFOV, high-frequency oscillatory ventilation; ICU, intensive care unit; IMV, intermittent mandatory ventilation; IVH, intraventricular hemorrhage; PDA, patent ductus arteriosus; PSV, pressure support ventilation; PSV + VG, pressure support ventilation with volume-guarantee ventilation; PTV, patient-triggered ventilation; RDS, respiratory distress syndrome; SIMV, synchronized intermittent mechanical ventilation; SIMV + PSV, synchronized intermittent mechanical ventilation with pressure support ventilation; SIMV + VG, synchronized intermittent mechanical ventilation with volume-guarantee ventilation; SIPPV + VG, synchronized intermittent positive pressure ventilation plus volume-guarantee ventilation; TCPL, time-cycled pressure-limited ventilation; V-C, volume-controlled (through adjusting the delivered tidal volume).

**Table 2:** **The 16 ventilation modes for infants with respiratory distress syndrome**

HFFIV	High-frequency flow interrupted ventilation
TCPL	Time-cycled pressure-limited ventilation
HFOV	High-frequency oscillatory ventilation
A/C + VG	Assist-control plus volume-guarantee ventilation
A/C	Assist-control ventilation
SIMV + VG	Synchronized intermittent mechanical ventilation with volume-guarantee ventilation
SIMV	Synchronized intermittent mechanical ventilation
A/C + VG + RM	Assist-control plus volume-guarantee ventilation with recruitment maneuver
PSV + VG	Pressure support ventilation with volume-guarantee ventilation
PSV	Pressure support ventilation
V-C	Volume-controlled (through adjusting the delivered tidal volume)
PTV	Patient-triggered ventilation
IMV	Intermittent mandatory ventilation
SIPPV + VG	Synchronized intermittent positive pressure ventilation plus volume-guarantee ventilation
HFJV	High-frequency jet ventilation
SIMV + PSV	Synchronized intermittent mechanical ventilation with pressure support ventilation

### Primary outcome

#### Mortality

**Figure 3:**
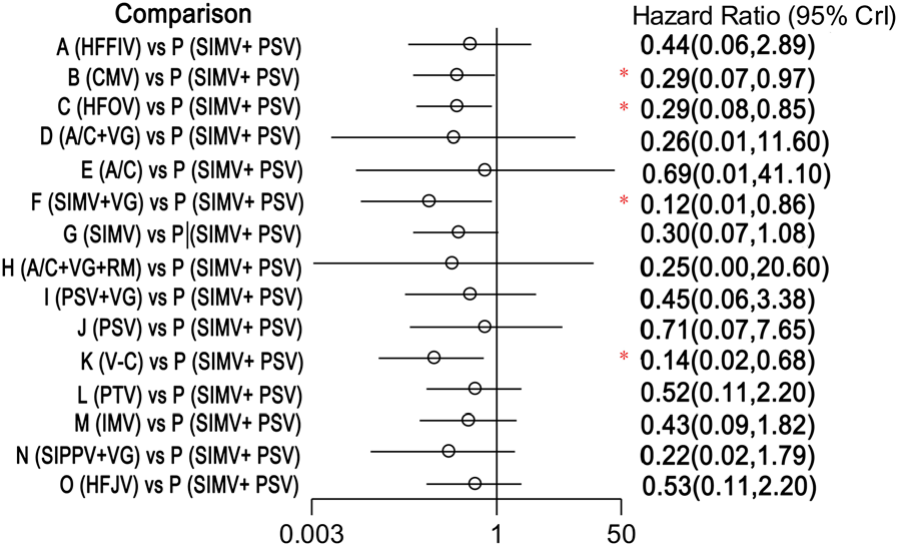
**Hazard ratios for death in the Bayesian network meta-analysis versus SIMV + PSV.** Hazard ratios are estimated from a fixed-effects Bayesian network meta-analysis. *95% confidence interval (CI) for the Bayesian network meta-analysis does not contain 1. A/C, assist-control ventilation; A/C + VG, assist-control plus volume-guarantee ventilation; A/C + VG + RM, assist-control plus volume-guarantee ventilation with recruitment maneuver; CMV, continuous mandatory ventilation; HFFIV, high-frequency flow interrupted ventilation; HFJV, high-frequency jet ventilation; HFOV, high-frequency oscillatory ventilation; IMV, intermittent mandatory ventilation; PSV, pressure support ventilation; PSV + VG, pressure support ventilation with volume-guarantee ventilation; PTV, patient-triggered ventilation; SIMV, synchronized intermittent mechanical ventilation; SIMV + PSV, synchronized intermittent mechanical ventilation with pressure support ventilation; SIMV + VG, synchronized intermittent mechanical ventilation with volume-guarantee ventilation; SIPPV + VG, synchronized intermittent positive pressure ventilation plus volume-guarantee ventilation; V-C, volume-controlled (through adjusting the delivered tidal volume).

**Figure 4:**
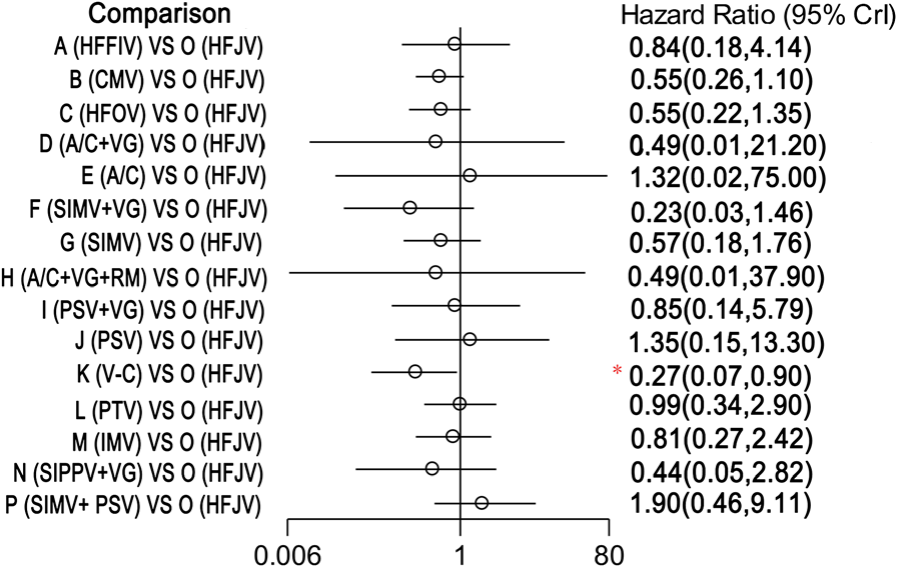
**Hazard ratios for death in the Bayesian network meta-analysis versus high-frequency jet ventilation (HFJV).** Hazard ratios are estimated from a fixed-effects Bayesian network meta-analysis. *95% confidence interval (CI) for the Bayesian network meta-analysis does not contain 1. A/C, assist-control ventilation; A/C + VG, assist-control plus volume-guarantee ventilation; A/C + VG + RM, assist-control plus volume-guarantee ventilation with recruitment maneuver; CMV, continuous mandatory ventilation; HFFIV, high-frequency flow interrupted ventilation; HFOV, high-frequency oscillatory ventilation; IMV, intermittent mandatory ventilation; PSV, pressure support ventilation; PSV + VG, pressure support ventilation with volume-guarantee ventilation; PTV, patient-triggered ventilation; SIMV, synchronized intermittent mechanical ventilation; SIMV + PSV, synchronized intermittent mechanical ventilation with pressure support ventilation; SIMV + VG, synchronized intermittent mechanical ventilation with volume-guarantee ventilation; SIPPV + VG, synchronized intermittent positive pressure ventilation plus volume-guarantee ventilation; V-C, volume-controlled (through adjusting the delivered tidal volume).

**Figure 5:**
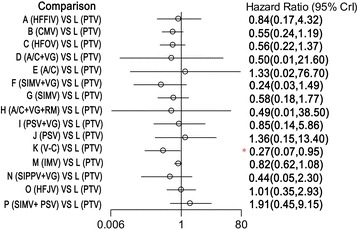
**Hazard ratios for death in the Bayesian network meta-analysis versus patient-triggered ventilation (PTV).** Hazard ratios are estimated from a fixed-effects Bayesian network meta-analysis. *95% confidence interval (CI) for Bayesian network meta-analysis does not contain 1. A/C, assist-control ventilation; A/C + VG, assist-control plus volume-guarantee ventilation; A/C + VG + RM, assist-control plus volume-guarantee ventilation with recruitment maneuver; CMV, continuous mandatory ventilation; HFFIV, high-frequency flow interrupted ventilation; HFJV, high-frequency jet ventilation; HFOV, high-frequency oscillatory ventilation; IMV, intermittent mandatory ventilation; PSV, pressure support ventilation; PSV + VG, pressure support ventilation with volume-guarantee ventilation; SIMV, synchronized intermittent mechanical ventilation; SIMV + PSV, synchronized intermittent mechanical ventilation with pressure support ventilation; SIMV + VG, synchronized intermittent mechanical ventilation with volume-guarantee ventilation; SIPPV + VG, synchronized intermittent positive pressure ventilation plus volume-guarantee ventilation; V-C, volume-controlled (through adjusting the delivered tidal volume).

All included studies reported information in regard to mortality and therefore were included in the network meta-analysis. One of these trials was a three-arm experiment, and the remaining trials were two-arm experiments. Compared with the synchronized intermittent mandatory ventilation (SIMV) + pressure support ventilation (PSV) mode, the time-cycled pressure-limited ventilation (TCPL), HFOV, SIMV + VG, and V-C modes were associated with a reduction in mortality in the infants with RDS. Specifically, the HRs (and 95% CIs) for the TCPL, HFOV, SIMV + VG, and V-C modes were 0.290 (0.071 to 0.972), 0.294 (0.080 to 0.852), 0.122 (0.014 to 0.858), and 0.139 (0.024 to 0.677), respectively (Figure 3). Compared with the high-frequency jet ventilation (HFJV) and patient-triggered ventilation (PTV) modes, the V-C mode was associated with a reduction in mortality in the infants with RDS, with HRs (95% CIs) of 0.267 (0.073 to 0.897) and 0.269 (0.070 to 0.951) relative to the HFJV and PTV modes, respectively (Figures 4 and 5).

Additional file 6 summarizes the rankings of the competing treatment modes with regard to mortality. The SIMV + VG mode was associated with the greatest potential to reduce mortality; the probability that this ventilation approach was most effective was 29.7% most likely; the V-C mode was the second-ranked ventilation approach, with a probability of 22.8% most likely. The A/C mode was most likely the worst approach with respect to mortality, and the SIMV + PSV approach was most likely to be ranked second or third from the bottom. Comparisons of the mortality results from traditional pairwise meta-analyses and the network meta-analysis did not suggest statistical inconsistencies between the direct and indirect evidence; moreover, none of the comparisons indicated statistical heterogeneity (Additional file 7).

The sensitivity analysis that excluded the articles by Salvo *et al*. [27] and Castoldi *et al*. [28] from the analysis did not change the reported results. Therefore, a different gestational age did not result in statistical heterogeneity in our network meta-analysis, and our final results included the articles by Salvo *et al*. [27] and Castoldi *et al*. [28], as previously described.

### Secondary outcomes

#### Patent ductus arteriosus

We identified eight trials with 584 participants that reported the incidences of PDA. However, we were able to include only 519 participants randomly assigned across five treatment modes from six trials [11,30,33,37-39] in our network meta-analysis because the incidence of PDA in two studies [10,28] could not be incorporated into the network analysis. The combined results of the direct and indirect comparisons demonstrated that these five ventilation modes exhibited no differences with respect to the incidences of PDA (Additional file 8). Furthermore, no statistical inconsistencies were identified between the direct and indirect comparisons, and none of the comparisons exhibited statistical heterogeneity (Additional file 9).

### Incidences of intraventricular hemorrhage (grade of at least III)

Eight articles [7,10,27,31,33,34,37,39] reported the incidences of IVH (grade of at least III) and involved nine ventilation modes. However, we were able to include only seven RCTs across seven ventilation modes in our network meta-analysis because the incidence of IVH (grade of at least III) in the article by Nafday *et al*. [37] could not be incorporated in the network analysis. The combined results of the direct and indirect comparisons demonstrated that these seven ventilation modes exhibited no differences in regard to the incidences of IVH (grade of at least III) (Additional file 10). No statistical inconsistencies were identified between the direct and indirect comparisons, and none of the comparisons exhibited statistical heterogeneity (Additional file 11).

### Other outcomes

Ten studies [10,11,29,31,32,34-36,39,41] reported the incidences of pneumothorax, eight studies [10-12,28,29,36,38,41] reported the incidences of BPD, eight studies [6,10,12,27,32,33,38,41] reported the length of hospital stay, and two studies [27,38] reported the ICU length of stay. Unfortunately, certain treatment measures were isolated from the remaining treatment measures in these studies; therefore, the aforementioned outcomes could not be examined via network meta-analysis.

## Discussion

The diversity and strength of a network are determined by the number of different interventions and comparisons of interventions that are available, how represented they are in the network, and how much evidence they carry. A severe imbalance in regard to the amount of evidence for each intervention may affect the power and reliability of the overall analysis. Random-effects meta-analysis models can accommodate unexplained heterogeneity for the available pairwise comparisons and often make the incoherence signals less prominent. None of the comparisons identified statistical heterogeneity in our network meta-analysis; thus, we chose a Bayesian fixed-effects model based on the guidelines of Dias *et al*. [21].

Because of the limitations of a traditional meta-analysis, Bhuta and Henderson-Smart [8] could only perform pairwise comparisons of the HFJV and CV modes; however, various modes of ventilation are used for the MV of infants with RDS, such as the intermittent mandatory ventilation (IMV), PTV, and HFJV modes. By taking advantage of a network meta-analysis, we compared all MV modes from acceptable RCTs for infants with RDS to date. To the best of our knowledge, the current investigation is the first application of a network meta-analysis to the MV modes of infants with RDS, and the results obtained from the current analyses provide suggestions for the treatment of infants with RDS.

The network meta-analysis results demonstrated that the TCPL, HFOV, SIMV + VG, and V-C modes were associated with a lower mortality rate in infants with RDS compared with the SIMV + PSV mode. Moreover, the V-C mode was associated with a lower mortality rate in infants with RDS than the HFJV and PTV modes. No differences were identified in regard to the TCPL, HFOV, SIMV, PSV + VG, or V-C modes with respect to the incidences of PDA.

We demonstrated that the SIMV + VG ventilation mode was the most successful at decreasing the mortality of infants with RDS but that the V-C mode ranked second. In addition, the A/C ventilation mode was associated with the worst in that it had the highest potential mortality rate of all ventilation modes, whereas the SIMV + PSV mode ranked second to last. But the probability ranking represents only a possibility without certainty; combining the direct and indirect evidence analysis on overall mortality has more reference significance.

VG and V-C ventilation are modes of volume-targeted ventilation that can ensure a constant target volume delivery via automatic adjustment of the peak inspiratory pressure from breath to breath on the basis of changes in pulmonary compliance (such as auto-weaning) [42,43]. Studies [12,44] have demonstrated that the VG ventilation mode has certain advantages, such as the prevention of lung atelectasis as well as the reduction of ventilation duration and the incidence of BPD and IVH, in infants with RDS; these advantages may occur through the previously discussed mechanism that can ensure the delivery of a constant tidal volume close to a physiological level via the adjustment of the peak inspiratory pressure (PIP) [42]. SIMV may reduce the duration of MV and oxygen dependency because it may also minimize oxygen therapy to prevent the development of severe BPD and retinopathy of prematurity (ROP) [23,45].

V-C may benefit from better lung recruitment and ventilation perfusion matching [11]. Furthermore, the limitation of excessive tidal volume and the automatic reduction of PIP may improve venous return and cardiac output, which further improves cerebral blood flow [46].

Lista *et al*. [36] demonstrated that pro-inflammatory cytokines are substantially increased and MV lasted longer in infants with RDS during the PSV ventilation mode. Moreover, pulmonary inflammation and oxygen toxicity are likely important predisposing factors in the development of chronic lung disease [36]. Because relatively few participants received the A/C ventilation mode, these results are more likely to be biased [47].

### Limitations

Our research has certain limitations. First, few experiments examined (and therefore few participants received) the A/C and A/C + VG + recruitment maneuver (RM) ventilation modes compared with the other modes. In addition, there was a lack of relevant treatment measures; thus, the meta-analysis results might be prone to bias. Future multicenter comparisons that involve large sample sizes and directly perform parallel comparisons of ventilation modes are needed to confirm our results. Second, because the results of the included experiments are incomplete, the network meta-analysis of the incidences of pneumothorax and BPD as well as the lengths of ICU and hospital stay was not conducted. Therefore, the results of the current investigation are relatively simple, and comprehensive and diverse conclusions cannot be provided. Third, the ventilation mode during the period of weaning may have an effect on the treatment of infants with RDS; however, given that the time for weaning is so short, we did not consider the ventilation mode during the period of weaning in our network meta-analysis. Finally, surfactant is an important drug for preterm infants with RDS. However, nearly every infant has used surfactant in our included literatures; thus, we also did not consider the use of surfactant in our analyses.

## Conclusions

Compared with the SIMV + PSV ventilation mode, the TCPL, HFOV, SIMV + VG, and V-C ventilation modes are associated with lower mortality.

## Key messages

- MV remains an essential and life-saving technique to care for preterm infants with RDS for whom non-invasive ventilation fails.
- We attempted to provide suggestions for the treatment of infants with RDS by taking advantage of a network meta-analysis.
- Compared with the SIMV + PSV ventilation mode, the TCPL, HFOV, SIMV + VG, and V-C ventilation modes are associated with lower mortality.

## Additional files

Additional file 1:
**Search strategy.**
Additional file 2:
**Model fit for mortality – results.**
Additional file 3:
**Model fit for patent ductus arteriosus (PDA) – results.**
Additional file 4:
**Model fit for intraventricular hemorrhage (IVH) (grade of at least III) – results.**
Additional file 5:
**Summary of excluded articles.**
Additional file 6:
**Rankings based on simulations of mortality.**
Additional file 7:
**The mortality effect estimates from a multiple treatment meta-analysis compared with the direct and indirect estimates, which were based on back-calculated and pair-wise meta-analyses.**
Additional file 8:
**The combined results of the direct and indirect comparisons of five ventilation modes in regard to the incidences of patent ductus arteriosus (PDA).**
Additional file 9:
**The patent ductus arteriosus (PDA) effect estimates from a multiple treatment meta-analysis compared with the direct and indirect estimates, which are based on back-calculated and pair-wise meta-analyses.**
Additional file 10:
**The combined results of the direct and indirect comparisons of seven ventilation modes with respect to the incidences of intraventricular hemorrhage (IVH) (grade of at least III).**
Additional file 11:
**The intraventricular hemorrhage (IVH) (grade of at least III) effect estimates from a multiple treatment meta-analysis compared with the direct and indirect estimates, which were based on back-calculated and pair-wise meta-analyses.**

## Abbreviations

A/C: assist-control ventilation
BPD: bronchopulmonary dysplasia
CI: confidence interval
DIC: deviance information criterion
HFJV: high-frequency jet ventilation
HFOV: high-frequency oscillatory ventilation
HR: hazard ratio
ICU: intensive care unit
IVH: intraventricular hemorrhage
MD: mean difference
MV: mechanical ventilation
OR: odds ratio
PDA: patent ductus arteriosus
PIP: peak inspiratory pressure
PSV: pressure support ventilation
PTV: patient-triggered ventilation
RCT: randomized controlled trial
RDS: respiratory distress syndrome
SIMV: synchronized intermittent mechanical ventilation
TCPL: time-cycled pressure-limited ventilation
V-C: volume-controlled
VG: volume-guarantee ventilation
