# Supplementary material for: Mechanical ventilation modes for respiratory distress syndrome in infants: a systematic review and network meta-analysis
Source: Crit Care. 2015 Mar 20;19(1):108. doi: 10.1186/s13054-015-0843-7 (PMC4391657; doi:10.1186/s13054-015-0843-7)
Supplement: Additional file 1: — Search strategy. [file 13054_2015_843_MOESM1_ESM.doc]

**Additional file 1. Search Strategy**

1. Search strategy for CENTRAL, The Cochrane Library

#1 MeSH descriptor: [[Respiratory Distress Syndrome, Newborn](http://www.ncbi.nlm.nih.gov/mesh/68012127)] explode all trees

#2 Respiratory Distress Syndrome:ti,ab,kw (Word variations have been searched)

#3 Acute Respiratory Distress Syndrome:ti,ab,kw (Word variations have been searched)

#4 RDS:ti,ab,kw (Word variations have been searched)

#5 #1 or #2 or #3 or #4

#6 MeSH descriptor: [Respiration, Artificial] explode all trees

#7 lung protective ventilation strategy:ti,ab,kw (Word variations have been searched)

#8 protective near ventilation:ti,ab,kw (Word variations have been searched)

#9 mechanical ventilation:ti,ab,kw (Word variations have been searched)

#10 LPVS:ti,ab,kw (Word variations have been searched)

#11 #7 or #8 or #9 or #10

#12 #5 and #11

2. Search strategy for EMBASE (OvidSP)

1. respiratory distress syndrome/ or respiratory distress/ or respiratory distress syndrome.mp. or acute respiratory failure.mp. or acute respiratory distress syndrome.mp. or rds.mp.

2. respiration, artificial/ or lung protective ventilation strategy/ or (protective adj3 ventilation).mp. or mechanical ventilation.mp. or LPVS.mp. [mp=title, abstract, subject headings, heading word, drug trade name, original title, device manufacturer, drug manufacturer, device trade name, keyword]

3. 1 and 2

4. (placebo.sh. or controlled study.ab. or random*.ti,ab. or trial*.ti,ab. or ((singl* or doubl* or trebl* or tripl*) adj3 (blind* or mask*)).ti,ab.) not (animals not (humans and animals)).sh.

5. 3 and 4

3. Search strategy for MEDLINE (OvidSP)

1. exp Respiratory Distress Syndrome, Newborn/ or respiratory distress/ or Acute Respiratory Distress Syndrome/ or respiratory distress syndrome.mp. or acute respiratory failure.mp. or acute respiratory distress syndrome.mp. or rds.mp.

2. exp Respiration, Artificial / or lung protective ventilation strategy / or (protective adj3 ventilation).mp. or mechanical ventilation or LPVS.mp.

3. 1 and 2

4. ((randomized controlled trial or controlled clinical trial).pt. or randomized.ab. or placebo.ab. or clinical trials as topic.sh. or randomly.ab. or trial.ti.) not (animals not (humans and animals)).sh.

5. 3 and 4

4. Search strategy for CINAHL (EBSCOhost)

S1 (MH “Respiratory Distress Syndrome, Newborn”) OR (MH “Respiratory Distress Syndrome”) OR TX RDS

S2 (MM “Respiration, Artificial”) OR TI (mechanical ventilation) OR TX lung protective ventilation strategy OR AB ( protective and ventilation ) OR TX LPVS

S3 S1 and S2

5. Search strategy for ISI Web of Science

#1 TS=(respiratory distress syndrome or acute respiratory distress syndrome or rds)

#2 TS=(artificial respiration) or TS=(mechanical ventilation) or TS=(protective SAME ventilation) or TS= LPVS

#3 TS=(random* or placebo* or multicenter* or prospective) or TS=(trail* SAME (clinical or controlled))

#4 #1 and #2 and #3
