# Supplementary material for: Mechanical ventilation modes for respiratory distress syndrome in infants: a systematic review and network meta-analysis
Source: Crit Care. 2015 Mar 20;19(1):108. doi: 10.1186/s13054-015-0843-7 (PMC4391657; doi:10.1186/s13054-015-0843-7)
Supplement: Additional file 3: — Model fit for patent ductus arteriosus (PDA) – results. [file 13054_2015_843_MOESM3_ESM.doc]

**Additional file 3.Model fit for PDA – results**

|  | **Mean deviance** | **Penalty (pD)** | **DIC** |
| --- | --- | --- | --- |
| **Fixed effects model** | **55.44895** | **9.93733** | **65.38628** |
|
| **Random effects model** | **55.7033** | **10.38841** | **66.09171** |
|

Mean deviance indicates the posterior mean of the residual deviance. pD indicates the effective number of parameters (leverage).DIC indicates the ’Deviance Information Criterion’. A lower Mean deviance and DIC indicates a better model fit, based on the above information, fixed-effect model is the preferred model.
