# Supplementary material for: Mechanical ventilation modes for respiratory distress syndrome in infants: a systematic review and network meta-analysis
Source: Crit Care. 2015 Mar 20;19(1):108. doi: 10.1186/s13054-015-0843-7 (PMC4391657; doi:10.1186/s13054-015-0843-7)
Supplement: Additional file 5: — Summary of excluded articles. [file 13054_2015_843_MOESM5_ESM.doc]

**Additional file 5. Summary of excluded articles**

| **Year** | **Authors** | **Exclued Reasons** |
| --- | --- | --- |
| 1998 | [Quinn MW](http://www.ncbi.nlm.nih.gov/pubmed?term=Quinn MW%5BAuthor%5D&cauthor=true&cauthor_uid=9713031) | crossover study |
| 1997 | Chen JY | patients do not match |
| 1996 | Samuels MP | patients do not match |
| 1993 | Ogawa Y | patients do not match |
| 1991 | OCTAVE Study Group | patients do not match |
| 1989 | [The HIFI Study Group.](http://www.ncbi.nlm.nih.gov/pubmed/2643039) | patients do not match |
| 1987 | [Han VK](http://www.ncbi.nlm.nih.gov/pubmed?term=Han VK%5BAuthor%5D&cauthor=true&cauthor_uid=3102211) | no relevant outcome |
| 1983 | Carlon GC | patients do not match |
| 1996 | Hawker F | communicate with the author |
| 1994 | Murai DT | patients do not match |
| 2003 | [Brunherotti MA](http://www.ncbi.nlm.nih.gov/pubmed?term=Brunherotti MA%5BAuthor%5D&cauthor=true&cauthor_uid=12973513) | crossover study |
| 1998 | Thome U | patients do not match |
| 2006 | Reyes ZC | patients do not match |
| 1999 | A Johnson | letters to the editor |
| 1999 | [Thome U](http://www.ncbi.nlm.nih.gov/pubmed?term=Thome U%5BAuthor%5D&cauthor=true&cauthor_uid=10393602) | patients do not match |
| 2000 | [Bauer K1](http://www.ncbi.nlm.nih.gov/pubmed?term=Bauer K%5BAuthor%5D&cauthor=true&cauthor_uid=10890665) | no relevant outcome |
| 2000 | [Esteban A](http://www.ncbi.nlm.nih.gov/pubmed?term=Esteban A%5BAuthor%5D&cauthor=true&cauthor_uid=10858404) | patients do not match |
| 2000 | [Rimensberger PC](http://www.ncbi.nlm.nih.gov/pubmed?term=Rimensberger PC%5BAuthor%5D&cauthor=true&cauthor_uid=10835058) | no control group |
| 2003 | [Craft AP](http://www.ncbi.nlm.nih.gov/pubmed?term=Craft AP%5BAuthor%5D&cauthor=true&cauthor_uid=12556921) | patients do not match |
| 2003 | [Dubois MJ](http://www.ncbi.nlm.nih.gov/pubmed?term=Dubois MJ%5BAuthor%5D&cauthor=true&cauthor_uid=12617746) | meeting report |
| 2003 | [Osborn DA](http://www.ncbi.nlm.nih.gov/pubmed?term=Osborn DA%5BAuthor%5D&cauthor=true&cauthor_uid=12970631) | patients do not match |
| 2002 | Johnson AH | patients do not match |
| 2004 | Varpula T | patients do not match |
| 2005 | [D'Angio CT](http://www.ncbi.nlm.nih.gov/pubmed?term=D'Angio CT%5BAuthor%5D&cauthor=true&cauthor_uid=16143747) | patients do not match |
| 2003 | Jian Li | patients do not match |
| 2005 | Oczenski W | patients do not match |
| 2005 | [Vento G](http://www.ncbi.nlm.nih.gov/pubmed?term=Vento G%5BAuthor%5D&cauthor=true&cauthor_uid=15717206) | patients do not match |
| 2005 | [Walsh MC](http://www.ncbi.nlm.nih.gov/pubmed?term=Walsh MC%5BAuthor%5D&cauthor=true&cauthor_uid=15973322) | no control group |
| 2005 | Xun Wu | patients do not match |
| 2006 | [Curley MA](http://www.ncbi.nlm.nih.gov/pubmed?term=Curley MA%5BAuthor%5D&cauthor=true&cauthor_uid=16616620) | patients do not match |
| 2000 | [Mrozek JD](http://www.ncbi.nlm.nih.gov/pubmed?term=Mrozek JD%5BAuthor%5D&cauthor=true&cauthor_uid=10613781) | no relevant outcome |
| 2008 | Chun-Jun Liu | no relevant outcome |
| 2008 | Meade MO | patients do not match |
| 2009 | [Moraes MA](http://www.ncbi.nlm.nih.gov/pubmed?term=Moraes MA%5BAuthor%5D&cauthor=true&cauthor_uid=18989547) | patients do not match |
| 2011 | Chu-Ming You | no control group |
| 2011 | [Lucangelo U](http://www.ncbi.nlm.nih.gov/pubmed?term=Lucangelo U%5BAuthor%5D&cauthor=true&cauthor_uid=22205035) | patients do not match |
| 2012 | Van Haperen1 M | meeting abstract |
| 2013 | [Amini E](http://www.ncbi.nlm.nih.gov/pubmed?term=Amini E%5BAuthor%5D&cauthor=true&cauthor_uid=23983995) | patients do not match |
| 2013 | [Cruces P](http://www.ncbi.nlm.nih.gov/pubmed?term=Cruces P%5BAuthor%5D&cauthor=true&cauthor_uid=23255291) | patients do not match |
| 2002 | Randolph AG | lack of data |
| 2011 | Grassino. E C | no control group |
| 1987 | [Froese AB](http://www.ncbi.nlm.nih.gov/pubmed?term=Froese AB%5BAuthor%5D&cauthor=true&cauthor_uid=3304021) | no control group |
| 1991 | Abbasi S | patients do not match |
| 1991 | [Hird MF](http://www.ncbi.nlm.nih.gov/pubmed?term=Hird MF%5BAuthor%5D&cauthor=true&cauthor_uid=1804948) | no relevant outcome |
| 2002 | Courtney SE | patients do not match |
| 2003 | [Cambonie G](http://www.ncbi.nlm.nih.gov/pubmed?term=Cambonie G%5BAuthor%5D&cauthor=true&cauthor_uid=14599072) | no relevant outcome |
| 2003 | Cheng C | not randomized trial |
| 1997 | [Keszler M](http://www.ncbi.nlm.nih.gov/pubmed?term=Keszler M%5BAuthor%5D&cauthor=true&cauthor_uid=9310511) | crossover study |
| 1998 | [Rettwitz-Volk W](http://www.ncbi.nlm.nih.gov/pubmed?term=Rettwitz-Volk W%5BAuthor%5D&cauthor=true&cauthor_uid=9506636) | crossover study |
| 1999 | [Plavka R](http://www.ncbi.nlm.nih.gov/pubmed?term=Plavka R%5BAuthor%5D&cauthor=true&cauthor_uid=10051081) | crossover study |
| 2005 | [Ben Jaballah N](http://www.ncbi.nlm.nih.gov/pubmed?term=Ben Jaballah N%5BAuthor%5D&cauthor=true&cauthor_uid=15480780) | not randomized trial |
| 2005 | [Samransamruajkit R](http://www.ncbi.nlm.nih.gov/pubmed?term=Samransamruajkit R%5BAuthor%5D&cauthor=true&cauthor_uid=16572737) | crossover study ,patients do not match |
| 2013 | [Wilson PT](http://www.ncbi.nlm.nih.gov/pubmed?term=Wilson PT%5BAuthor%5D&cauthor=true&cauthor_uid=23164308) | no control group |
| 2000 | Simma B | no control group |
| 2006 | [Ben Jaballah N](http://www.ncbi.nlm.nih.gov/pubmed?term=Ben Jaballah N%5BAuthor%5D&cauthor=true&cauthor_uid=17001556) | patients do not match |
| 2006 | Lista G | no control group |
| 2006 | Reyes ZC | patients do not match |
| 2009 | Singh J | second research |
| 2013 | Pinzon AD | patients do not match |
| 2013 | Chowdhury O, | patients do not match |
|  | S Al-Alaiyan1 | no relevant outcome |
