# Supplementary material for: Mechanical ventilation modes for respiratory distress syndrome in infants: a systematic review and network meta-analysis
Source: Crit Care. 2015 Mar 20;19(1):108. doi: 10.1186/s13054-015-0843-7 (PMC4391657; doi:10.1186/s13054-015-0843-7)
Supplement: Additional file 6: — Rankings based on simulations of mortality. [file 13054_2015_843_MOESM6_ESM.doc]

**Additional file 6. Rankings based on simulations in terms of mortality**.

|  |  | |  | |  | |  | |  | |  |
| --- | --- | --- | --- | --- | --- | --- | --- | --- | --- | --- | --- |
|  | [,1] | | [,2] | | [,3] | | [,4] | | [,5] | | [,6] |
| A | 5.70E-02 | | 7.43E-02 | | 7.73E-02 | | 8.77E-02 | | 8.91E-02 | | 8.30E-02 |
| B | 5.00E-06 | | 4.50E-05 | | 6.75E-04 | | 3.65E-03 | | 1.30E-02 | | 3.51E-02 |
| C | 1.67E-06 | | 2.10E-04 | | 1.39E-03 | | 5.24E-03 | | 1.48E-02 | | 3.41E-02 |
| D | 6.27E-03 | | 8.06E-02 | | 1.08E-01 | | 5.90E-02 | | 4.65E-02 | | 4.06E-02 |
| E | 2.77E-01 | | 1.31E-01 | | 7.30E-02 | | 5.15E-02 | | 3.99E-02 | | 3.33E-02 |
| F | 2.05E-03 | | 4.57E-03 | | 6.91E-03 | | 9.96E-03 | | 1.31E-02 | | 1.57E-02 |
| G | 5.62E-04 | | 4.50E-03 | | 1.21E-02 | | 2.61E-02 | | 4.35E-02 | | 6.31E-02 |
| H | 8.55E-02 | | 9.17E-02 | | 8.10E-02 | | 4.82E-02 | | 3.73E-02 | | 3.22E-02 |
| I | 2.09E-02 | | 9.28E-02 | | 9.82E-02 | | 8.67E-02 | | 8.74E-02 | | 8.45E-02 |
| J | 2.04E-01 | | 1.41E-01 | | 9.88E-02 | | 9.34E-02 | | 7.67E-02 | | 5.95E-02 |
| K | 7.33E-05 | | 4.75E-04 | | 1.29E-03 | | 2.62E-03 | | 4.65E-03 | | 7.72E-03 |
| L | 2.77E-02 | | 7.30E-02 | | 1.11E-01 | | 1.40E-01 | | 1.52E-01 | | 1.43E-01 |
| M | 2.32E-03 | | 1.83E-02 | | 4.74E-02 | | 8.00E-02 | | 1.10E-01 | | 1.30E-01 |
| N | 1.70E-02 | | 2.62E-02 | | 3.11E-02 | | 3.85E-02 | | 4.34E-02 | | 4.77E-02 |
| O | 3.03E-02 | | 7.59E-02 | | 1.03E-01 | | 1.29E-01 | | 1.41E-01 | | 1.30E-01 |
| P | 2.69E-01 | | 1.85E-01 | | 1.49E-01 | | 1.39E-01 | | 8.77E-02 | | 6.17E-02 |
|  | [,7] | | [,8] | | [,9] | | [,10] | | [,11] | | [,12] |
| A | 7.91E-02 | | 7.13E-02 | | 6.27E-02 | | 6.02E-02 | | 5.83E-02 | | 5.42E-02 |
| B | 6.65E-02 | | 1.07E-01 | | 1.41E-01 | | 1.61E-01 | | 1.62E-01 | | 1.37E-01 |
| C | 6.50E-02 | | 1.07E-01 | | 1.50E-01 | | 1.75E-01 | | 1.69E-01 | | 1.36E-01 |
| D | 3.59E-02 | | 3.36E-02 | | 3.22E-02 | | 3.10E-02 | | 3.32E-02 | | 4.49E-02 |
| E | 2.97E-02 | | 2.69E-02 | | 2.46E-02 | | 2.50E-02 | | 3.22E-02 | | 4.56E-02 |
| F | 1.91E-02 | | 2.21E-02 | | 2.62E-02 | | 3.27E-02 | | 4.77E-02 | | 7.57E-02 |
| G | 8.49E-02 | | 1.04E-01 | | 1.17E-01 | | 1.26E-01 | | 1.27E-01 | | 1.11E-01 |
| H | 2.85E-02 | | 2.66E-02 | | 2.48E-02 | | 2.46E-02 | | 2.77E-02 | | 3.64E-02 |
| I | 7.30E-02 | | 6.51E-02 | | 6.03E-02 | | 5.85E-02 | | 5.85E-02 | | 5.64E-02 |
| J | 4.86E-02 | | 4.10E-02 | | 3.71E-02 | | 3.49E-02 | | 3.33E-02 | | 3.35E-02 |
| K | 1.18E-02 | | 1.71E-02 | | 2.44E-02 | | 3.63E-02 | | 6.16E-02 | | 1.06E-01 |
| L | 1.17E-01 | | 8.82E-02 | | 6.12E-02 | | 3.85E-02 | | 2.36E-02 | | 1.44E-02 |
| M | 1.34E-01 | | 1.22E-01 | | 1.04E-01 | | 8.41E-02 | | 6.25E-02 | | 4.62E-02 |
| N | 5.07E-02 | | 5.08E-02 | | 5.14E-02 | | 5.45E-02 | | 6.61E-02 | | 7.98E-02 |
| O | 1.16E-01 | | 9.16E-02 | | 6.67E-02 | | 4.76E-02 | | 3.08E-02 | | 1.93E-02 |
| P | 3.99E-02 | | 2.64E-02 | | 1.68E-02 | | 1.06E-02 | | 6.51E-03 | | 3.79E-03 |
|  | | [,13] | | [,14] | | [,15] | | [,16] | |  | |
| A | | 5.19E-02 | | 4.71E-02 | | 3.28E-02 | | 1.40E-02 | |  | |
| B | | 1.02E-01 | | 5.67E-02 | | 1.44E-02 | | 9.22E-04 | |  | |
| C | | 8.97E-02 | | 4.13E-02 | | 1.06E-02 | | 1.10E-03 | |  | |
| D | | 6.86E-02 | | 1.02E-01 | | 1.70E-01 | | 1.08E-01 | |  | |
| E | | 5.93E-02 | | 9.64E-02 | | 4.02E-02 | | 1.46E-02 | |  | |
| F | | 1.13E-01 | | 1.30E-01 | | 1.84E-01 | | 2.97E-01 | |  | |
| G | | 9.16E-02 | | 6.26E-02 | | 2.26E-02 | | 2.01E-03 | |  | |
| H | | 5.32E-02 | | 8.40E-02 | | 1.12E-01 | | 2.06E-01 | |  | |
| I | | 5.51E-02 | | 5.04E-02 | | 3.64E-02 | | 1.59E-02 | |  | |
| J | | 3.32E-02 | | 2.93E-02 | | 2.26E-02 | | 1.31E-02 | |  | |
| K | | 1.38E-01 | | 1.67E-01 | | 2.28E-01 | | 1.94E-01 | |  | |
| L | | 7.11E-03 | | 2.43E-03 | | 4.47E-04 | | 4.33E-05 | |  | |
| M | | 3.37E-02 | | 1.89E-02 | | 6.97E-03 | | 1.09E-03 | |  | |
| N | | 8.98E-02 | | 1.04E-01 | | 1.17E-01 | | 1.32E-01 | |  | |
| O | | 1.15E-02 | | 5.80E-03 | | 1.76E-03 | | 2.78E-04 | |  | |
| P | | 2.30E-03 | | 1.28E-03 | | 4.93E-04 | | 7.17E-05 | |  | |

The numbers in the table represent the probability that each treatment is best (rank 1), the second highest (rank 2), etc. Rank probabilities sum to 1, both within a rank over treatments and within a treatment over ranks.
