# Supplementary material for: Mechanical ventilation modes for respiratory distress syndrome in infants: a systematic review and network meta-analysis
Source: Crit Care. 2015 Mar 20;19(1):108. doi: 10.1186/s13054-015-0843-7 (PMC4391657; doi:10.1186/s13054-015-0843-7)
Supplement: Additional file 8: — The combined results of the direct and indirect comparisons of five ventilation modes in regard to the incidences of patent ductus arteriosus (PDA). [file 13054_2015_843_MOESM8_ESM.doc]

**Additional file 8.** The combined results of the direct and indirect comparisons of 5 ventilation modes with respect to the incidences of PDA.

Comparison

Odds Ratio (95% CrI)

TCPL vs V-C

1.11 (0.54, 2.27)

HFOV vs V-C

1.01 (0.423, 2.42)

SIMV vs V-C

0.62 (0.0353, 9.93)

PSV+VG vs V-C

0.529 (0.0473, 4.98)

1

0.03

10

Comparison

Odds Ratio (95% CrI)

TCPL vs PSV+VG

2.09 (0.25, 21)

HFOV vs PSV+VG

1.91 (0.242, 18.4)

SIMV vs PSV+VG

1.18 (0.243, 6.01)

V-C vs PSV+VG

1.89 (0.201, 21.2)

1

0.2

30

Comparison

Odds Ratio (95% CrI)

TCPL vs SIMV

1.77 (0.106, 31)

HFOV vs SIMV

1.63 (0.106, 26.6)

PSV+VG vs SIMV

0.844 (0.154, 4.4)

V-C vs SIMV

1.58 (0.0821, 30.5)

1

0.08

40

Comparison

Odds Ratio (95% CrI)

TCPL vs HFOV

1.09 (0.577, 2.04)

SIMV vs HFOV

0.612 (0.0375, 9.45)

PSV+VG vs HFOV

0.518 (0.0532, 4.5)

V-C vs HFOV

0.964 (0.337, 2.71)

1

0.03

10

Odds ratios for the incidences of PDA in the Bayesian network meta-analysis direct and indirect comparisons of 5 ventilation modes. CI= confidence interval for Bayesian network meta-analysis. Odds ratios (ORs) estimated from a fixed effects, Bayesian network meta-analysis. ^*^ 95% CI does not contain 1.
