# Supplementary material for: Mechanical ventilation modes for respiratory distress syndrome in infants: a systematic review and network meta-analysis
Source: Crit Care. 2015 Mar 20;19(1):108. doi: 10.1186/s13054-015-0843-7 (PMC4391657; doi:10.1186/s13054-015-0843-7)

**Additional file 9.** The PDA effect estimates from a multiple treatment meta-analysis compared with the direct and indirect estimates, which were based on back-calculated, and pair-wise meta-analyses. Direct and indirect estimates of effect and the corresponding Bayesian ‘I^2^’ for inconsistency were calculated. And the‘I^2^’from Pooled pair-wise meta-analysis for heterogeneity were also calculated.


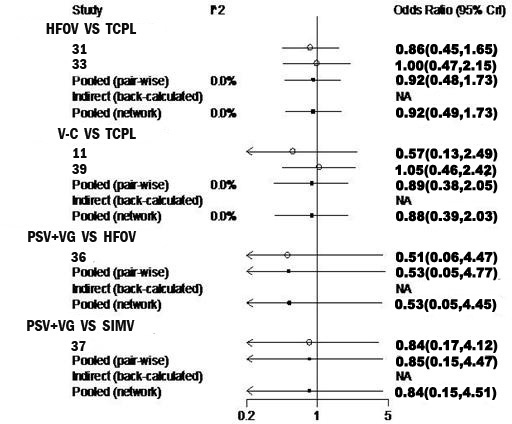

Supplement: Additional file 9: — The patent ductus arteriosus (PDA) effect estimates from a multiple treatment meta-analysis compared with the direct and indirect estimates, which are based on back-calculated and pair-wise meta-analyses. [file 13054_2015_843_MOESM9_ESM.doc]
