# Supplementary material for: Mechanical ventilation modes for respiratory distress syndrome in infants: a systematic review and network meta-analysis
Source: Crit Care. 2015 Mar 20;19(1):108. doi: 10.1186/s13054-015-0843-7 (PMC4391657; doi:10.1186/s13054-015-0843-7)
Supplement: Additional file 10: — The combined results of the direct and indirect comparisons of seven ventilation modes with respect to the incidences of intraventricular hemorrhage (IVH) (grade of at least III). [file 13054_2015_843_MOESM10_ESM.doc]

**Additional file 10.** The combined results of the direct and indirect comparisons of 7 ventilation modes with respect to the incidences of IVH（grade≥Ⅲ）


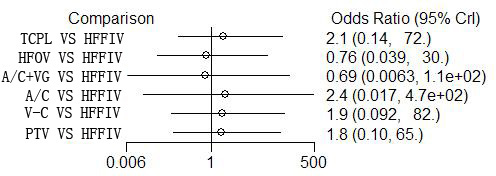


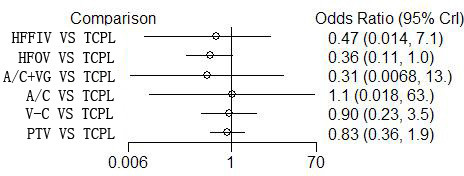


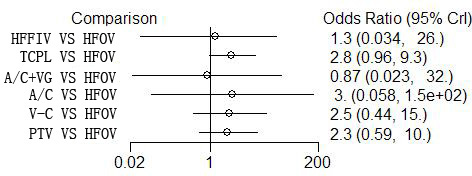


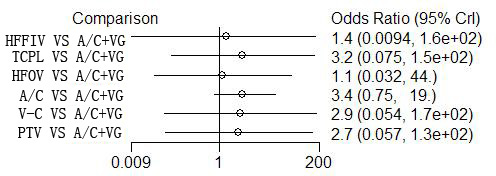


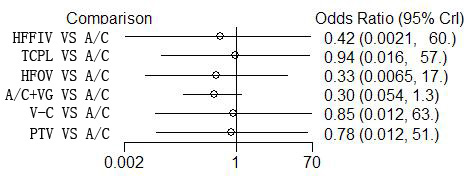


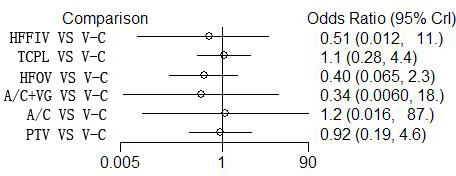


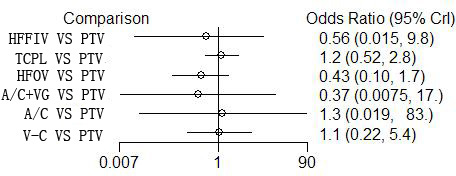


Odds ratios for the incidences of IVH（grade≥Ⅲ） in Bayesian network meta-analysis direct and indirect comparisons of 7 ventilation modes. CI= confidence interval for Bayesian network meta-analysis. Odds ratios (ORs) estimated from a fixed effects, Bayesian network meta-analysis. ^*^ 95% CI does not contain 1.
